# Supplementary material for: LymphoAtlas: a dynamic and integrated phosphoproteomic resource of TCR signaling in primary T cells reveals ITSN2 as a regulator of effector functions
Source: Mol Syst Biol. 2020 Jul 3;16(7):e9524. doi: 10.15252/msb.20209524 (PMC7333348; doi:10.15252/msb.20209524)
Supplement: Supplementary file 1 — Appendix [file MSB-16-e9524-s001.pdf]

Appendix

Table of Content:

Appendix Figure S1: Molecular assessment of ITSN2 targeted T cells.

Appendix Figure S2: LymphoAtlas, a website to explore phosphoproteomic data in primary mouse T cells.

Appendix Table S1: List of antibodies used for mass-cytometry analysis.

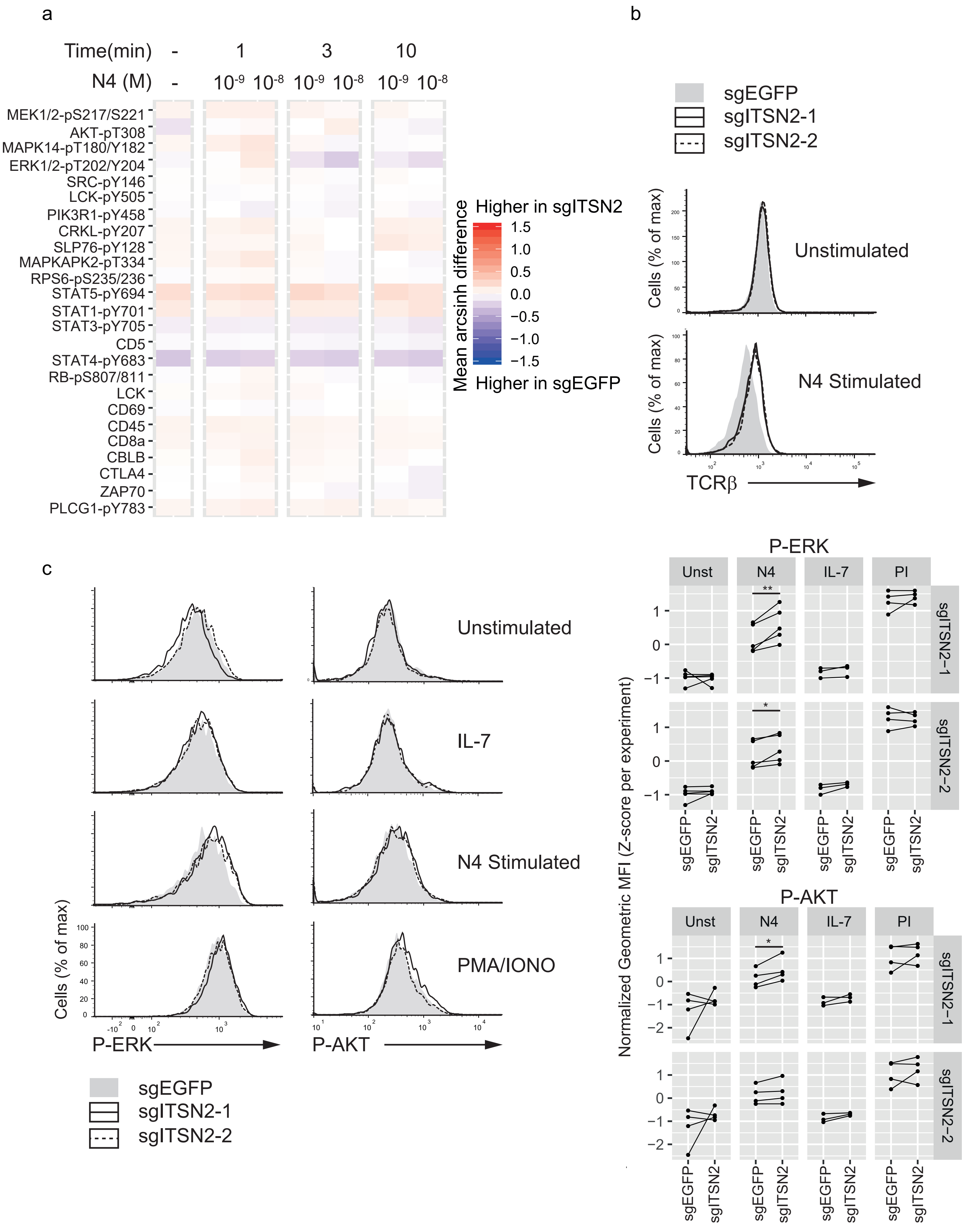

a
Time(min)
-
1
3
10
N4 (M)
-
10-9 10-8 10-9 10-8 10-9 10-8
MEK1/2-pS217/S221
AKT-pT308
MAPK14-pT180/Y182
ERK1/2-pT202/Y204
SRC-pY146
LCK-pY505
PIK3R1-pY458
CRKL-pY207
SLP76-pY128
MAPKAPK2-pT334
RPS6-pS235/236
STAT5-pY694
STAT1-pY701
STAT3-pY705
CD5
STAT4-pY683
RB-pS807/811
LCK
CD69
CD45
CD8a
CBLB
CTLA4
ZAP70
PLCG1-pY783
Mean arcsinh difference
Higher in sgITSN2
1.5
1.0
0.5
0.0
−0.5
−1.0
−1.5
Higher in sgEGFP
b
sgEGFP
sgITSN2-1
sgITSN2-2
Cells (% of max)
Unstimulated
N4 Stimulated
TCRβ
c
Unstimulated
IL-7
N4 Stimulated
PMA/IONO
Cells (% of max)
P-ERK
P-AKT
sgEGFP
sgITSN2-1
sgITSN2-2
P-ERK
Unst
N4
IL-7
PI
sgITSN2−1
sgITSN2−2
sgEGFP
sgITSN2
Normalized Geometric MFI (Z-score per experiment)
P-AKT

Appendix Figure S1: Molecular assessment of ITSN2 targeted T cells. Cas9-EGFP OT-I $CD8^+$ T cells were transfected with control sgRNA (sgEGFP) or sgRNA targeting *Itsn2* (sgITSN2-1 or sgITSN2-2). **a** Heatmap showing the difference between control cells and *Itsn2* targeted cells left unstimulated (-) or stimulated for 1, 3 or 10 min with N4 tetramers (1 or 10 nM) and analyzed by single-cell mass cytometry using antibodies specific for the indicated total proteins and phosphoproteins (color scale corresponds to the difference between mean of arcsinh transformed fluorescence intensities of two independent transfections with sgITSN2-1 and sgITSN2-2). **b** Cells transfected with the indicated type of sgRNA were left unstimulated or stimulated with N4 peptide (10 nM) for 4 h, subsequently stained with an anti-TCRβ antibody and analyzed by flow cytometry. (**c**) Similar cells as in (b) were left untreated or treated with IL-7 (5ng/ml), N4 peptide (10 nM) or with PMA/ Ionomycin (PMA/IONO) for 6 h and subsequently stained with anti-ERK1/2-pY204/T202 and anti-AKT-pS473 antibodies and analyzed by flow cytometry. A representative FACS profile is shown (left). Results of n=5 (p-ERK), n=4 (p-AKT) independent experiments (right). Comparison between sgEGFP and sgITSN2 conditions were performed using a paired t-test. (*, $P \leq 0.05$, **, $P \leq 0.01$).

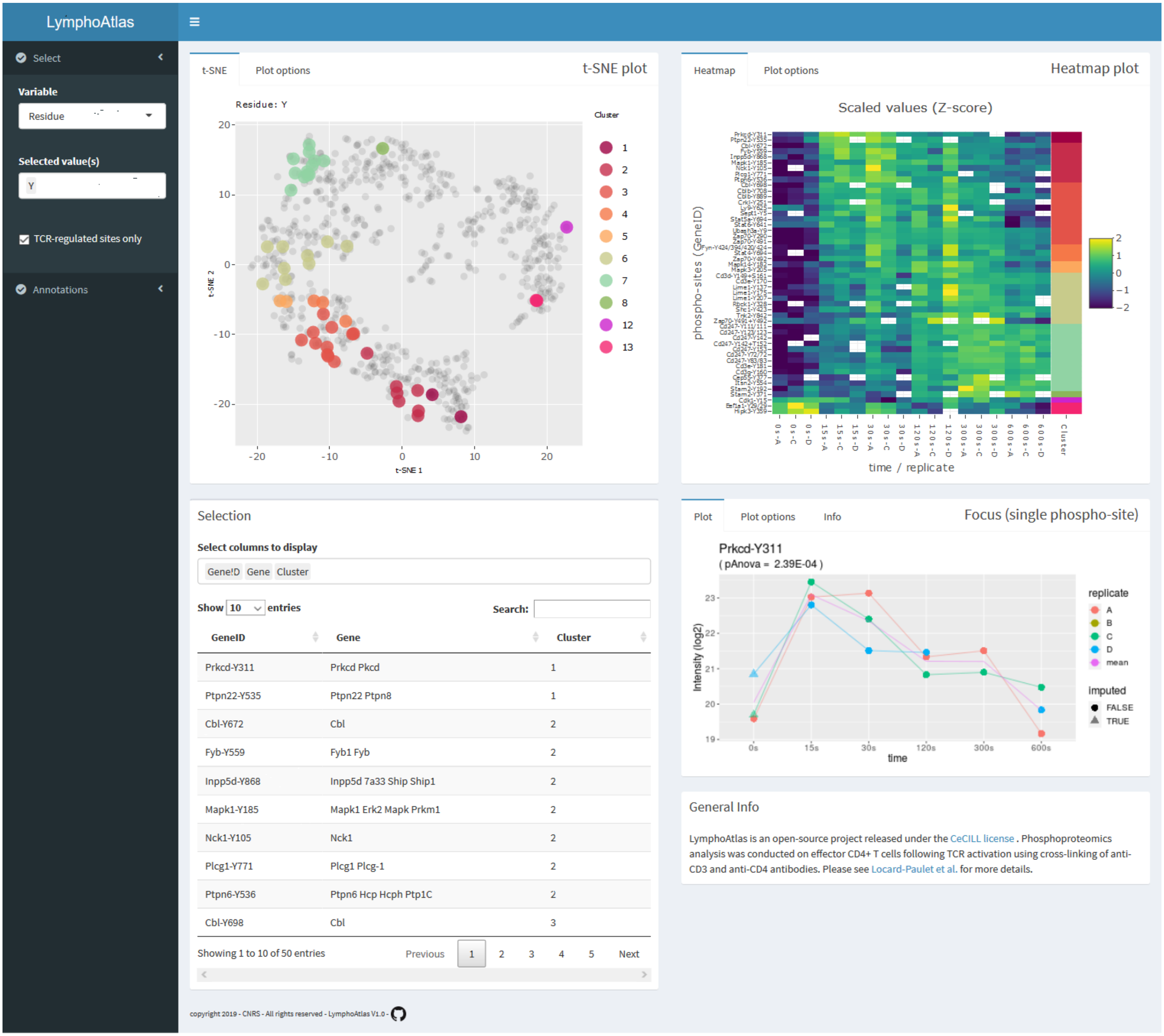

Appendix Figure S2: LymphoAtlas, a website to explore phosphoproteomic data in primary mouse T cells. Screen shot of the LymphoAtlas website (https://bmm-lab.github.io/LymphoAtlas).

Name	Target antigen	Clone	Tag	Titre	Mix	Short term stim	Long term stim	Comments
TCRb	TCR beta	H57-597	115In		Surface		x	
CD69	CD69	H1.2F3	145Nd		Surface		x	
CD44	CD44	IM7	171Yb		Surface		x	
CD45	CD45	30F11	89Y	100	Surface	x	x	
CD69	CD69	H1.2F3	143Nd	100	Surface	x		
CD5	CD5	53-7.3	160Gd	100	Surface	x	x	
CD8a	CD8a	53-6.7	168Er	100	Surface	x		
LCK	LCK	D88	145Nd	50	Surface	x		
ZAP70	ZAP70	99F2	147Sm	50	Surface	x		
CBLB	CBL-B	D3C12	155Gd	50	Surface	x		
CTLA4	CTLA-4	UC10-4B9	154Sm	100	Surface	x		
CFSE	CFSE	Polyclonale (Southern #6400-01)	174Yb	100	Intra	x		
SRC-pY146	pSRC Family [Y416]	D49G4	141Pr	100	Intra	x		
STAT4-pY683	pSTAT4 [Y683]	38/p-Stat4	148Nd	100	Intra	x		
ERK1/2-pT202/Y204	pERK1/2 [T202/Y204]	E10	149Sm	50	Intra	x	x	
STAT5-pY694	pSTAT5 [Y694]	47	150Nd	400	Intra	x		
PIK3R1-pY458	pPI-3K p85 [Y458]/p55 [Y199]	Polyclonal (CST cat#4228)	151Eu	100	Intra	x	x	
CRKL-pY207	pCRKL [Y207]	K30-391.50.80	152Sm	3200	Intra	x		
STAT1-pY701	pSTAT1 [Y701]	58D6	153Eu	400	Intra	x		
MAPK14-pT180/Y182	pp38 MAPK [T180/Y182]	D3F9	156Gd	400	Intra	x	x	
STAT3-pY705	pSTAT3 [Y705]	4/P-Stat3	158Gd	100	Intra	x		
MAPKAPK2-pT334	pMAPKAPK2	27B7	159Tb	400	Intra	x	x	
LCK-pY505	pLCK [Y505]	4/LCK-Y505	162Dy	400	Intra	x		Fluidigm (Cat# 3162004A)
PLCG1-pY783	pPLCg1 [Y783]	D6M9S	163Dy	100	Intra	x		Fluidigm (but removed from Cat#)
RPS6-pS235/236	p-p70 S6 kinase [S235/236]	D57.2.2E	164Dy	200	Intra	x	x	
SLP76-pY128	pSLP76 [Y128]	J141-668.36.58	165Ho	800	Intra	x	x	
RB-pS807/811	pRb [S807/811]	J112-906	166Er	100	Intra	x	x	
MEK1/2-pS217/S221	pMEK1/2 [S217/S221]	41G9	167Er	100	Intra	x	x	
AKT-pT308	pAKT [T308]	D25E6	169Tm	200	Intra	x	x	
ZAP70-pY319	pZAP70 [Y319] / pSYK [Y352]	17a	171Yb	100	Intra	x		
SHIP-1-pT1020	pSHIP-1 [T1020]	Polyclonal (CST cat#3941)	175Lu	200	Intra	x		
NFKB-pS933	pNF-kB p105 [S933]	18E6	176Yb	200	Intra	x	x	
CD4	CD4	RM4-5	172Yb	100	Surface	x		

Appendix Table S1: List of antibodies used for mass-cytometry analysis.
