# Supplementary material for: LymphoAtlas: a dynamic and integrated phosphoproteomic resource of TCR signaling in primary T cells reveals ITSN2 as a regulator of effector functions
Source: Mol Syst Biol. 2020 Jul 3;16(7):e9524. doi: 10.15252/msb.20209524 (PMC7333348; doi:10.15252/msb.20209524)
Supplement: Supplementary file 2 — Expanded View Figures PDF [file MSB-16-e9524-s002.pdf]

Expanded View Figures

Figure EV1. Description and quality control of the phosphoproteomic data set.

A Immunoblot of equal protein amounts from total lysates of primary mouse $CD4^+$ T cells left unstimulated (–) or stimulated for the indicated times. Global tyrosine phosphorylation was probed with antibody against phosphorylated tyrosine (anti-P-Tyr). Phosphorylation of proteins was assessed using phospho-specific antibodies, as indicated on the right. Anti-VAV1 was used as loading control.

B Phosphosites identified in this study (biological replicates A–D). * Determined from the "evidence.txt" tables (MaxQuant search FDR ≤ 0.01). **Number of phosphorylated sites (or combination of sites in the case of multiply phosphorylated peptides) determined from MaxQuant "Phospho (STY)Sites.txt" tables. Classes 1, 2, 3, and 4 correspond to a phospholocalization score of > 75%; 50% < ≤ 75%; 25% < ≤ 50%; and ≤ 25%, respectively.

C Histogram of the number of phosphosites per protein that were identified (left) and significantly regulated (right) in the phosphoproteome.

D Protein lengths against the number of phosphosites identified per protein.

E Log_{10}-transformed relative abundances (iBAQ) of the proteins in the proteome (Voisinne *et al*, 2019) against the number of phosphosites identified per protein in the phosphoproteome.

F Histogram of the log_{10}-transformed relative abundances (iBAQ) of the proteins in the proteome (gray). The subset of these proteins that are phosphorylated in the phosphoproteome are indicated in red. Dashed lines: median values for each population.

G UniProt keywords enriched (hypergeometric test *P*-value ≤ 0.05, fold change ≥ 1.5, number of annotated proteins ≥ 2) in the set of phosphoproteins compared with the set of all proteins identified in the $CD4^+$ T cell proteome. Fold changes and *P*-values (*P = 0) are indicated for each term. Keyword terms highlighted in red are not enriched if the same analysis is restricted to phosphosites detected in the unstimulated condition.

Data information: The protein Titin (35,213 amino acids) is omitted in (D) and (E), and the 2 proteins with more than 30 phosphosites (Srrm1: 40 sites; Srrm2: 100 sites) are omitted in (C–E).

© 2020 The Authors

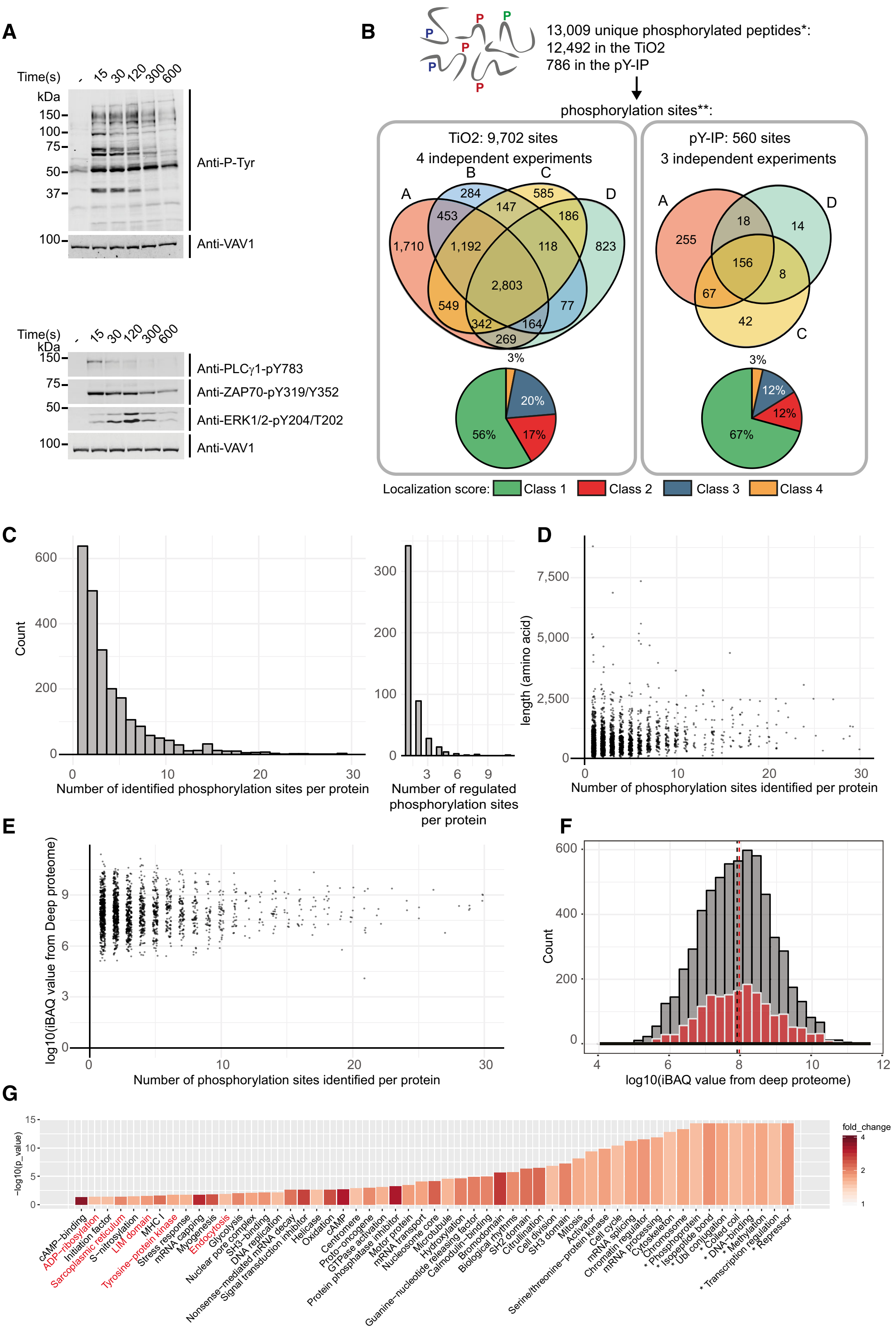
A
Time(s)
15 30 120 300 600
kDa
150
100
75
50
37
Anti-P-Tyr
100
Anti-VAV1
Time(s)
kDa
150
75
50
100
Anti-PLCγ1-pY783
Anti-ZAP70-pY319/Y352
Anti-ERK1/2-pY204/T202
Anti-VAV1
B
13,009 unique phosphorylated peptides*:
12,492 in the TiO2
786 in the pY-IP
phosphorylation sites**:
TiO2: 9,702 sites
4 independent experiments
pY-IP: 560 sites
3 independent experiments
Localization score: Class 1 Class 2 Class 3 Class 4
C
Count
Number of identified phosphorylation sites per protein
Number of regulated phosphorylation sites per protein
D
length (amino acid)
Number of phosphorylation sites identified per protein
E
log10(iBAQ value from Deep proteome)
Number of phosphorylation sites identified per protein
F
Count
log10(iBAQ value from deep proteome)
G
−log10(p_value)
fold_change

Figure EV1.

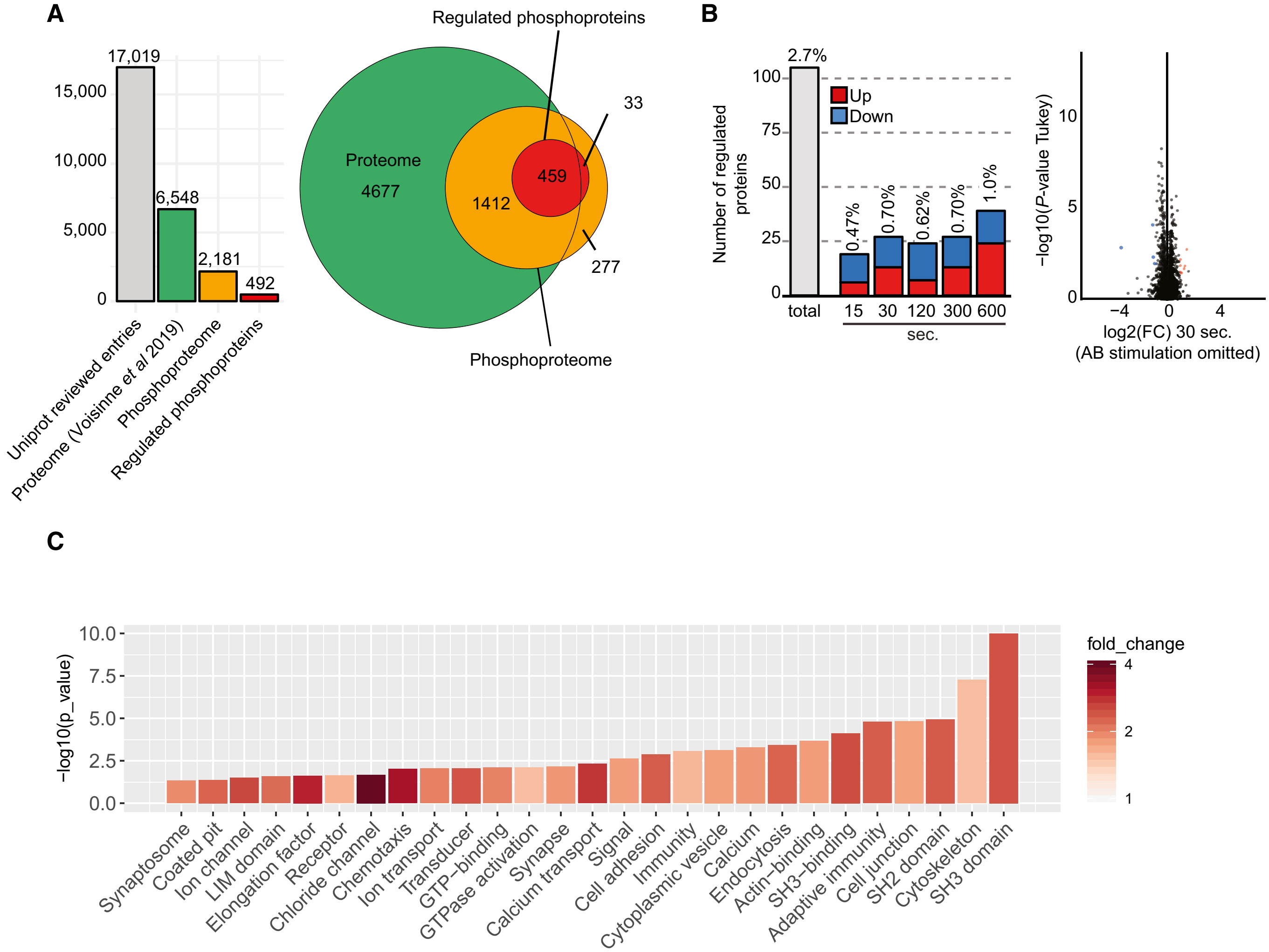

Figure EV2. Statistical and functional analysis of the phosphoregulations upon TCR activation.

A Left: number of unambiguously identified proteins across the following data sets: UniProt reviewed entries (www.uniprot.org); a proteome of $CD4^+$ T cells (Voisinne *et al*, 2019) (PXD012826); the phosphoproteome and the set of TCR-regulated phosphoproteins. Right: repartition of unambiguously identified proteins across the proteome, the phosphoproteome, and the set of TCR-regulated phosphoproteins. The area of the circles in the Euler diagram illustrates the relative number of proteins per data set without being strictly proportional.

B Output of the statistical analysis of protein abundances during TCR stimulation. Number of proteins significantly regulated upon TCR stimulation presented in bar plots for each time point next to the total number of regulated proteins across the entire time course (total). The corresponding percentage of the proteome that is regulated is indicated above the bars. Right panel: representative volcano plot presenting the statistical significance distribution against the log_2-transformed fold change between 30 s (maximum number of regulated proteins) and the unstimulated control. For each condition, proteins were considered significantly up-regulated (red) or down-regulated (blue) when displaying a corrected *P*-value ≤ 0.05 (ANOVA test) and an absolute log_2-transformed fold change ≥ 1 (see Materials and Methods for more detailed information).

C Enriched UniProt–keywords (hypergeometric test *P*-value ≤ 0.05, fold change ≥ 1.5, number of annotated proteins ≥ 2) in the set of regulated phosphosites compared with the set of all identified phosphosites. Fold changes and *P*-values are indicated for each term.

 © 2020 The Authors

Figure EV3. Dynamics of phosphorylation and selected functional modules induced by TCR stimulation.

A Dynamics and cluster distribution of regulated phosphotyrosines, phospho–serines, and phosphothreonines.

B Illustration of TCR-regulated phosphosites within the polycomb-repressive complex 1 (PRC1) (Ub: ubiquitination; Ac: acetylation). The corresponding log_2-transformed MS intensities measured upon TCR activation for each of the biological replicates are shown below. Box plot elements: Center line corresponds to median, box limits correspond to the first and third quartiles, and whiskers indicate variability from Q1–1.5. IQR to Q3 + 1.5 IQR.

C Short-time TCR stimulation induces FOXO3 phosphorylation and NFATC2 dephosphorylation and promotes nucleus exit or entry, respectively. $CD4^+$ T cells left unstimulated (—) or stimulated for 2 and 5 min with anti-CD3 plus anti-CD4 antibodies were subjected to nuclear/cytoplasmic fractionation before immunoblot analysis with antibodies specific for NFATC2 and FOXO3. Arrows indicate phosphorylation (P) and dephosphorylation (deP) forms of the transcription factors. Lamin-B2 and GAPDH are used to control purity of nuclear and cytoplasmic extracts.

D t-SNE plot highlighting phosphosites associated with the UniProt keywords "Protein biosynthesis" and "Translation regulation". Dot transparency is scaled according to the *P*-value corresponding to the local enrichment of the annotation term (hypergeometric test, see method for more detailed information).

E Left: Dynamics of TCR-regulated phosphosites associated with the UniProt keywords "Protein biosynthesis" and "Translation regulation" (dots color-coded by cluster). Right: Schematic representation of the translational initiation and elongation complexes. TCR-regulated phosphosites are represented as small dots color-coded by cluster.

© 2020 The Authors

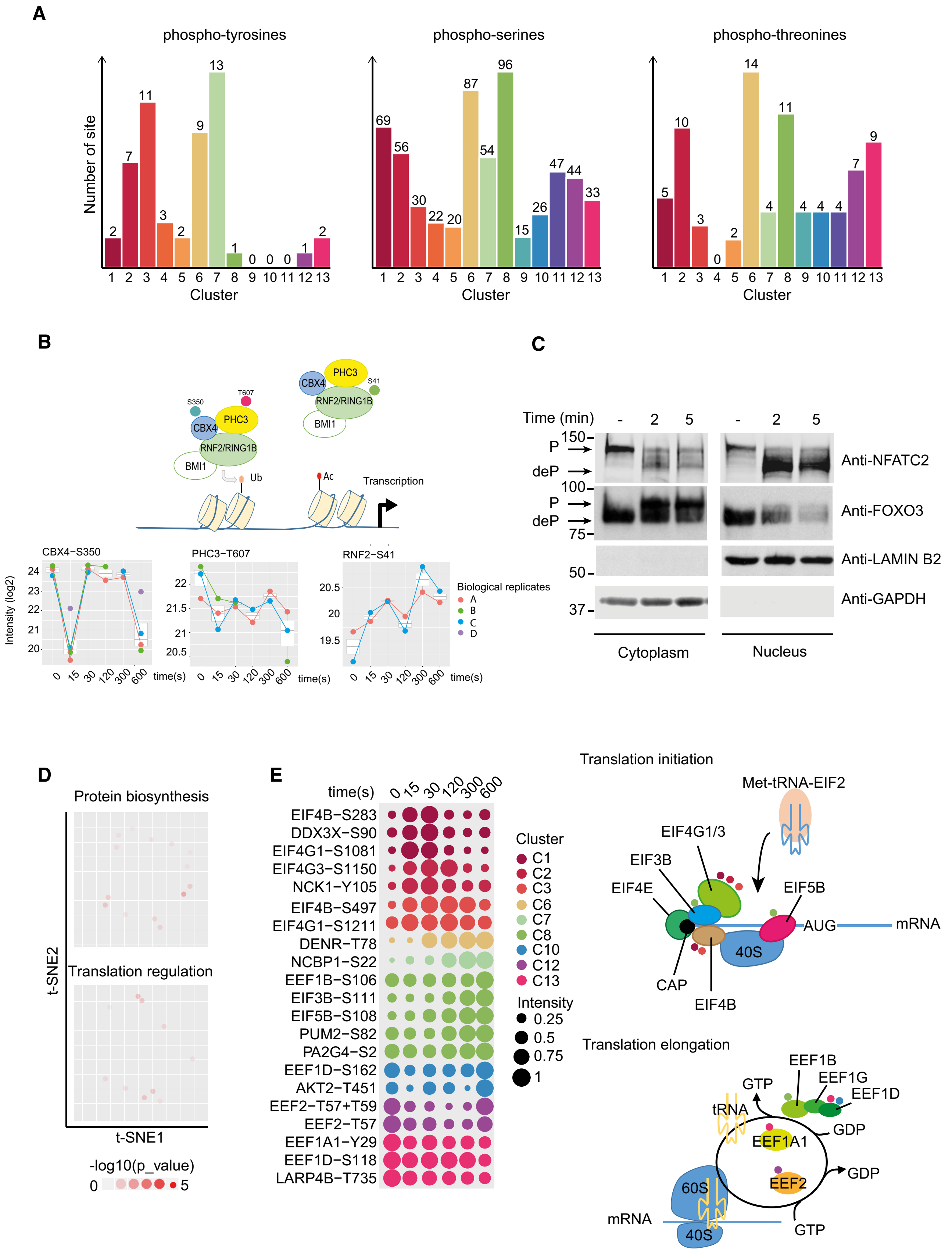

Figure EV3.

© 2020 The Authors

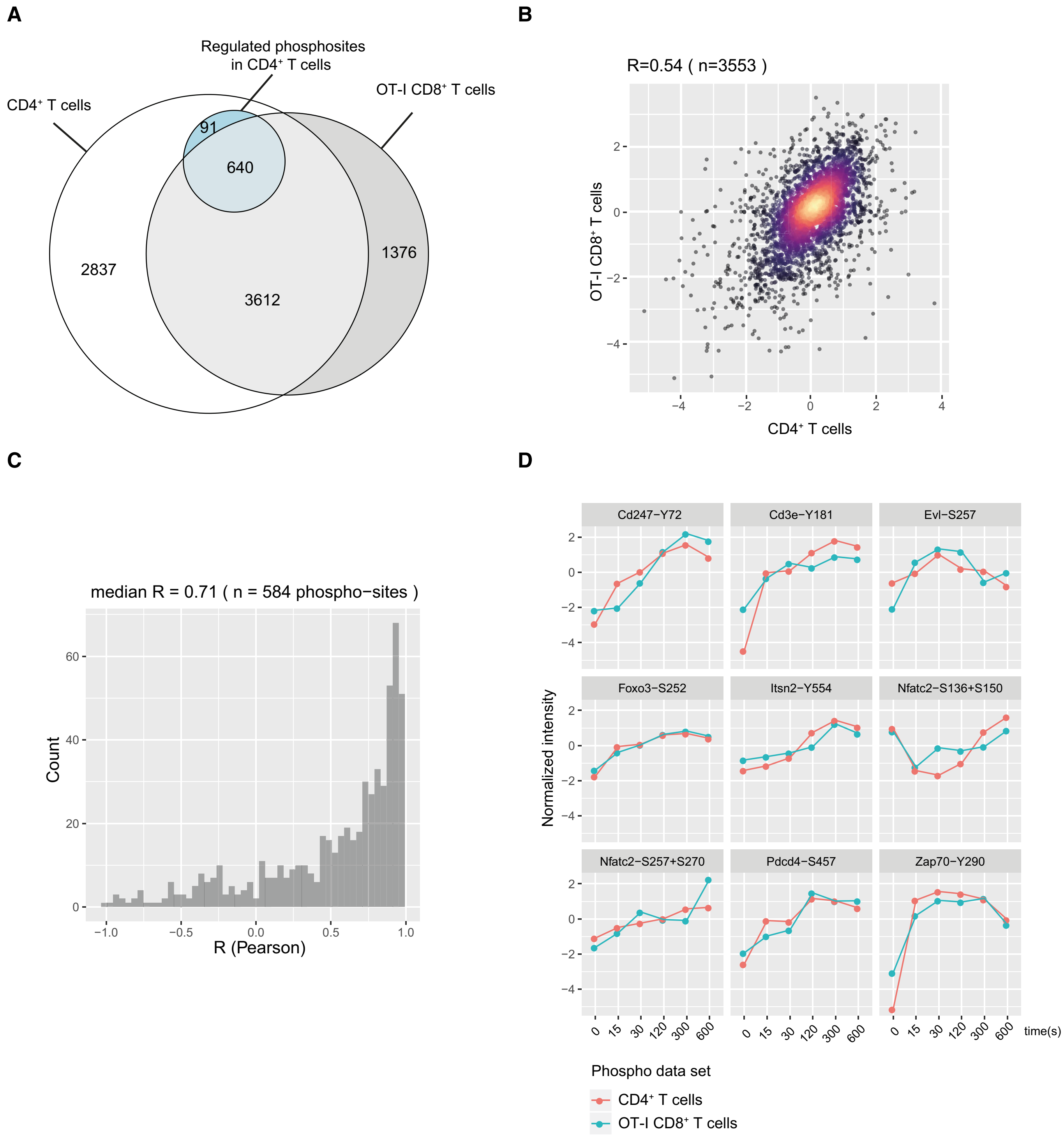

Figure EV4. Comparative analysis of phosphoproteomes performed with OT-I CD8⁺ and CD4⁺ T cells.

A Euler diagram indicating the number of phosphosites identified (3612) and regulated (640) upon TCR stimulation in CD4⁺ and OT-I CD8⁺ T cells.

B Comparison of phosphosite intensities between CD4⁺ and OT-I CD8⁺ T cells. Only sites regulated upon TCR stimulation in CD4⁺ T cells and identified in OT-I CD8⁺ T cells were considered. Log_2-transformed phosphosite intensities with imputed missing values were normalized across biological replicates using the mean intensity and subsequently averaged for each condition of stimulation. Pearson correlation coefficient $R = 0.54$.

C Distribution of Pearson's correlation coefficients across phosphosites regulated upon TCR stimulation in CD4⁺ T cells and identified in OT-I CD8⁺ T cells. Phosphosite intensities were normalized as in (B). The correlation coefficient was computed only for phosphosites with intensity values available in both data sets for a minimum of four stimulatory conditions ($n = 584$).

D Overlay dynamics of selected phosphosites in CD4⁺ and OT-I CD8⁺ T cells.

© 2020 The Authors

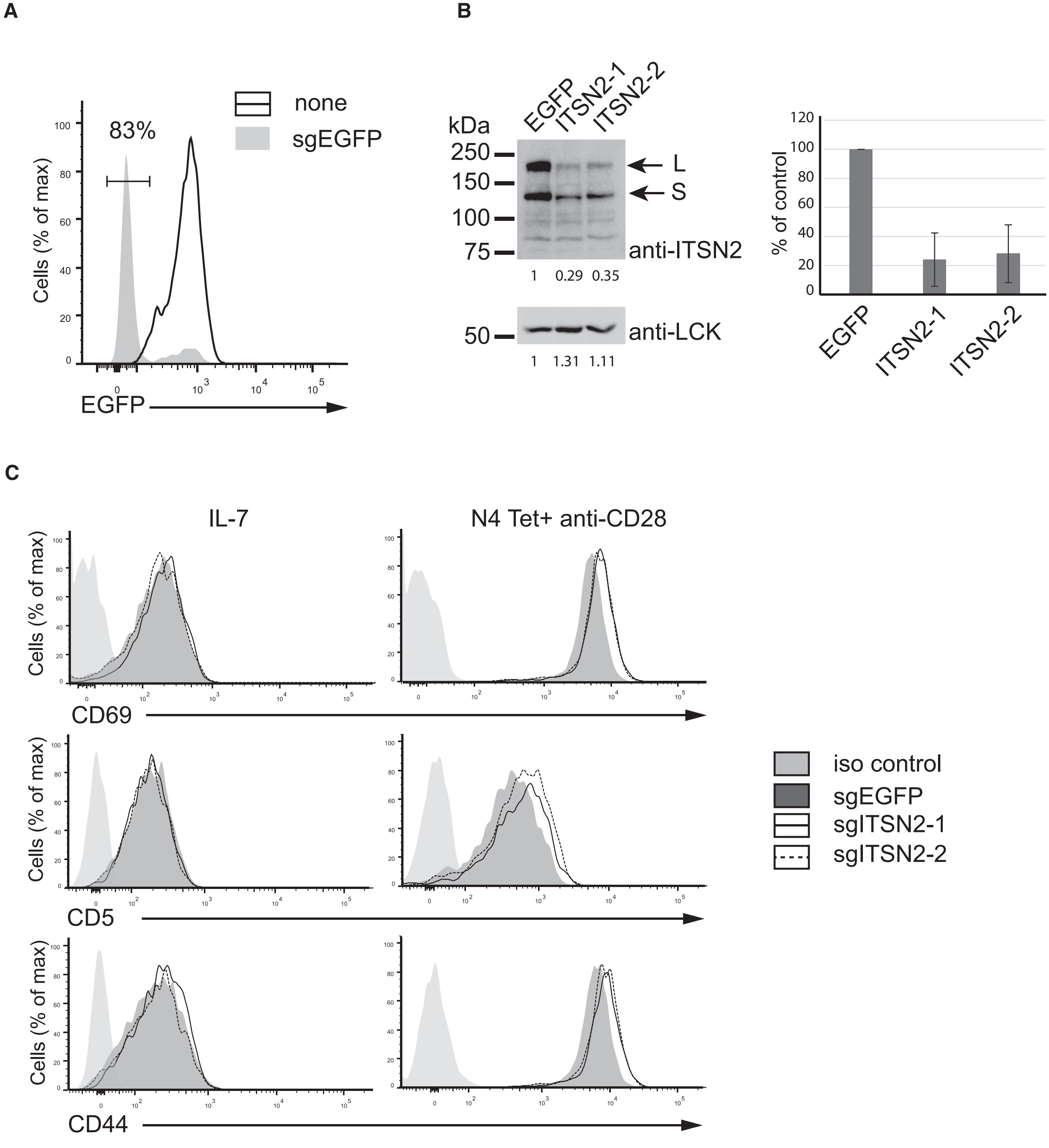

Figure EV5. ITSN2 inactivation by the CRISPR/Cas9 system in primary mouse T cells.

Cas9-EGFP OT-I CD8$^+$ T cells were transfected with control sgRNA (sgEGFP) or with two different sgRNA targeting *Itsn2* (sgITSN2-1 and sgITSN2-2).

A EGFP expression was assessed by flow cytometry in cells transfected with sgEGFP or without guide (none).

B Equal amounts of total lysates from cells transfected with sgEGFP or sgITSN2 were analyzed by immunoblot using anti-ITSN2 or anti-LCK antibodies. The arrows indicate short and long ITSN2 isoforms. Data are presented as mean ± SD from three independent experiments.

C Transfected cells were stimulated for 48 h with N4 peptide MHC tetramers in the presence of soluble anti-CD28 antibody or with IL-7 as control. Surface expression of CD69 and CD5 in cells was analyzed by flow cytometry. A representative FACS profile is shown.

 © 2020 The Authors
